# Establishing the prediction models for recurrence and progression of T1G3 bladder urothelial carcinoma

**DOI:** 10.7150/jca.35866

**Published:** 2019-10-11

**Authors:** Song Chen, Mengxin Lu, Tianchen Peng, Yejinpeng Wang, Xuefeng Liu, Yu Xiao, Xinghuan Wang

**Affiliations:** 1 Department of Urology, Zhongnan Hospital of Wuhan University, Wuhan, 430071, China; 2 Human Genetics Resource Preservation Center of Wuhan University, Wuhan, 430071, China; 3 Human Genetics Resource Preservation Center of Hubei Province, Wuhan, 430071 China; 4 Medical Research Institute, Wuhan University, Wuhan, 430071, China; 5 Department of Pathology, Lombardi Comprehensive Cancer Center, Georgetown University Medical School, Washington DC, USA; 6 Department of Biological Repositories, Zhongnan Hospital of Wuhan University, Wuhan, 430071, China

**Keywords:** Prediction models, recurrence, progression, T1G3, bladder urothelial carcinoma

## Abstract

We aim to determine clinical recurrence and progression risk factors of T1G3 bladder cancer (BCa), and to establish recurrence and progression prediction models. 5-year follow-up records of 106 T1G3 BCa patients from January 2012 to December 2016 were analyzed for recurrence and progression. Two-sample T-test, Chi-square test, Mann-Whitney test, Kaplan-Meier curves, Cox univariate and multivariate analyses were performed to determine the independent risk factors. Effective prognostic nomograms were established to provide individualized prediction, and the calibration curves were founded to evaluate the agreements of the predicted probability with the actual observed probability. Receiver operating characteristic (ROC) curves were generated for the recurrence and progression prediction models. The stability of prediction models was validated with an external cohort included 61 T1G3 BCa patients. Of the 106 T1G3 BCa patients, 77 were males (72.6%) and 29 were females (27.4%), with median age 70 years. Within 5 years, recurrence was identified in 67 cases (63.2%), and progression was identified in 31 cases (29.2%). The results showed that large size of tumor, multifocal tumors, recrudescent tumor, non-BCG perfusion therapy were the independent risk factors for recurrence, and large size of tumor, multifocal tumors, recrudescent tumor, concomitant carcinoma in situ (CIS) were the independent risk factors for progression. However, no evidence shown that tumor location or operative method was independent risk factors for recurrence and progression. Based on the results of Cox regression analyses, the independent risk factors were used to establish the prediction nomograms to calculate the recurrence and progression probability of each T1G3 BCa patient. Calibration curves, ROC curves and external validation displayed that the nomograms had great value of prediction.

## Introduction

An estimated 429,000 new cases of bladder cancer (BCa) were diagnosed each year in 2012, with 165,000 deaths per year in the world 1. And it is the fifth most common malignancy in men and 12th most common malignancy in women worldwide 2. Among these newly diagnosed patients, more than 70% of them are diagnosed with non-muscle-invasive bladder cancer (NMIBC) 3. Currently, despite the progress in diagnostic techniques and the improvement in surgical and nonsurgical therapies, bladder cancer has a high recurrence rate risk (ranging from 50 to 90 % of cases) and the prognosis of muscle-invasive bladder cancer (MIBC) has remained poor 2.

High-stage (T1) and high-grade (G3) have been repeatedly reported as important risk factors for NMIBC recurrence and progression in a number of publications 4-7, on account of the poor prognosis and the high recurrence rate of T1G3 BCa patients, the optimal treatment choice for these patients remains controversial. It has been reported that the 5-year progression-free survival rate of T1G3 BCa patients treated with transurethral resection of bladder tumor (TURBT) and intravesical Bacillus Calmette-Guérin (BCG)/chemotherapy was in the range of 60-80% 6, 8, while the T1G3 BCa patients with immediate radical cystectomy were reported with a 65-85% 5-year progression-free survival rate 5, 9, indicating that there was no much difference in prognosis between the two treatment approaches. In addition, Shahin O, *et al.* reported that after treated with TURBT and intravesical BCG therapy, about 30% of T1G3 BCa patients never had recurred, and 30% of patients need defer radical cystectomy, while another third finally died of metastatic disease 10. Given the variability of T1G3 BCa patients' outcomes, prediction of T1G3 recurrence and progression is particularly important to rightly identify high-risk patients.

According to the literatures, several adverse prognostic features have been associated with a high risk of BCa recurrence and progression, including concomitant carcinoma in situ (CIS), multifocal tumors, lymphovascular invasion, female sex, older age and large size of tumor (>3 cm) 11-13. Based on these factors, the EORTC risk tables and the CUETO scoring model were established to calculate the probability of recurrence and progression, and to improve the predictive accuracy of identifying high-risk patients 5, 14. However, most of these prediction models were based on different tumor stage and grade of NMIBC patients, and had a number of limitations for prediction of T1G3 patients with worse prognosis. In the current study, a retrospective analysis of 106 patients with T1G3 bladder cancer in our hospital was performed, to determine the recurrence risk factors of these patients, including large size of tumor, multifocal tumors, recrudescent tumor, Non-BCG perfusion therapy, as well as progression risk factors, including large size of tumor, multifocal tumors, recrudescent tumor and concomitant CIS. In addition, on the basis of our cox regression analyses, nomograms were constructed to calculate the probability of each T1G3 patient recurrence and progression directly, providing an evidence for clinical decision to balance surgical risks and tumor prognosis.

## Material and methods

### Study patients

This study contained a development cohort and a validation cohort. The development cohort included 106 patients with T1G3 bladder cancer at the Department of Urology, Zhongnan Hospital of Wuhan University from January 2012 to December 2016. All patients had undergone TURBT or partial cystectomy and accepted perfusion therapy post operation; the perfusion drugs included BCG, epirubicin, pyirubicin, hydroxycamptothecin and gemcitabine. The tumor stage and tumor grade of each patient was assessed according to the diagnostic criteria of 'Chinese diagnosis and treatment of urological diseases Guide'. Currently, the 1973 and the 2004 WHO classification co-exist. Several studies have compared the two classification methods, indicating that that WHO1973 grade cannot be replaced by the WHO2004 classification in NMIBC guidelines 15-17. In this study, 1973 classification standard (grade 1, 2, 3) was used as the grading system of bladder cancer. The patient follow-up protocol was performed with surveillance cystoscopy at 3-month intervals for the initial 2 years, and from the third year cystoscopy was performed every six months. The validation cohort included 61 patients with T1G3 BCa at the Department of Urology, Xiangyang Central Hospital from January 2013 to December 2018. The collection methods of clinical, pathological, follow-up data were as same as the development cohort. The clinical information was acquired by retrospective review of all patient medical records and approved by the Ethics Committee at Zhongnan Hospital of Wuhan University (approval number: 2015029). Informed consent was provided by all subjects. All procedures in this study were done in accordance with the ethical standards of the institutional and/or national research committee. In addition, all methods used for analysis in this study were carried out in accordance with the approved guidelines and regulations of the Department of Biological Repositories at Zhongnan Hospital of Wuhan University.

### Inclusion criteria

Patients were enrolled in this study if they met all the following criteria: (i) the T1G3 patients; (ii) patients who underwent surgery and perfusion therapy; (iii) had a complete and detailed clinical, pathological, follow-up data record.

### Exclusion criteria

Patients meeting any of the following criteria were excluded: (i) metastatic bladder cancer or merge other tumors; (ii) patients who did not undergo perfusion; (iii) patients who did not undergo surgery; (iv) any incomplete clinical, pathological or follow-up data.

### Outcomes and study design

All subjects were diagnosed with T1G3 bladder cancer by pathological examination. The outcomes were the recurrence or/and progression of T1G3 BCa within 5 years. The recurrence and progression were defined with surveillance cystoscopy and pathological examination. “Recurrence” was defined as the reappearance of bladder tumors after resection of primary tumors. “Progression” was defined that the recrudescent tumor was more malignant in clinical stage or pathological grade than the previous lesion.

### Statistical analysis

Continuous variables were depicted as averages, medians and ranges. Age was compared by two-sample t test. Chi-square test was performed for gender, smoking history, multifocal, past medical history, concomitant CIS, perfusion therapy and operative method. Mann-Whitney test was used for tumor location and tumor size. Cox univariate and multivariate analyses were performed to determine the independent recurrence and progression risk factors of T1G3 bladder urothelial carcinoma. Kaplan-Meier curves were generated to estimate 5-year recurrence survival and progression survival, and the log-rank test was used to assess survival differences among subgroups. Nomograms were generated based on cox regression analyses. The calibration curves were founded to evaluate the agreements of the nomogram-predicted probability with the actual observed probability. Receiver operating characteristic (ROC) curves were generated for the recurrence and progression prediction models, and the areas under the curves (AUC) were calculated. The stability of prediction models was confirmed with the validation cohort. SPSS 16.0 was used to perform all statistical analyses. Nomograms and calibration curves were generated with R version 3.5.0 and p value <0.05 was considered significant.

## Results

### Patient characteristics

In development cohort, a total of 1158 BCa patients were recorded hospitalized in our center from January 2012 to December 2016, 106 patients with T1G3 BCa of them (9.2%, 106/1158) were included. The median follow-up time was 31.8 months (range 2.6-67.4 months). 58 (54.7%) patients were followed up for more than 2 years, and 8 (7.5%) patients more than 5 years. As shown in table 1, recurrence was identified in 67 cases (63.2%). In these recurrence cases, 29 (27.4%) recrudesced within 12 months, while the recurrence time of 38 (35.8%) patients was more than 12 months. In addition, 38 (35.8%) recrudesced once, 29 (27.4%) recrudesced for multiple times (≥ twice). Progression was observed in 31 cases (29.2%), and 11 (10.4%) patients progressed within 12 months, while the progression time of 20 (18.9%) patients was more than 12 months. In the cases of death 17 (16.0%), 6 (5.7%) patients died within 24 months.

The detailed clinical parameters of enrolled patients in development cohort and validation cohort were presented in table 2, there was no significant difference in clinical parameters between the two cohorts (all p>0.05).

### Correlation analysis between recurrence, progression and clinicopathological factors of T1G3 bladder urothelial carcinoma

Table 3 listed the clinicopathological factors of T1G3 bladder cancer recurrence. Two-sample t test result showed that the old age was a risk factor of T1G3 BCa recurrence (p=0.047). Chi-square test results showed that T1G3 BCa recurrence was associated with gender (p=0.035), multifocal (p=0.018), past medical history (p=0.024) and perfusion therapy (p=0.028). Mann-Whitney test results indicated that the tumor size (p=0.031) were significant influencing factors of T1G3 BCa recurrence. Whereas no significant association was seen between T1G3 bladder cancer recurrence and several factors in our study, including smoking history, tumor location, concomitant CIS and operative method.

Additionally, to explore the progression factors of T1G3 bladder cancer, table 4 listed the clinicopathological factors of T1G3 bladder cancer progression. It showed that T1G3 BCa progression was associated with tumor size (p=0.026), multifocal (p=0.017), past medical history (p=0.045) and concomitant CIS (p=0.044).

### Cox univariate and multivariate analyses for T1G3 bladder urothelial carcinoma recurrence and progression

Cox univariate and multivariate analyses were performed to determine the independent recurrence and progression risk factors of T1G3 bladder urothelial carcinoma. Cox univariate analysis showed that tumor size (HR: 2.173; 95%CI: 1.412 - 3.368; p=0.014), multifocal (HR: 1.627; 95%CI: 1.128 - 3.095; p=0.035), past medical history (HR: 2.147; 95%CI: 1.237 - 4.058; p=0.042) and perfusion therapy (HR: 0.768; 95%CI: 0.341 - 0.922; p=0.048) were the influencing factors of T1G3 BCa recurrence. In agreement with univariate analysis results, cox multivariate analysis also demonstrated that large size of tumor (HR: 2.461; 95%CI: 1.358-3.975; p=0.021), multifocal tumors (HR: 2.524; 95%CI: 1.510-4.139; p<0.001), recrudescent tumor (HR: 3.069; 95%CI: 1.064-6.488; p=0.009) were the independent risk factors for T1G3 bladder cancer recurrence, and perfusion therapy with BCG (HR: 0.642; 95%CI: 0.289-0.864; p=0.012) was an independent protective factor for T1G3 bladder cancer recurrence (Table 5).

Similarly, table 6 showed the cox univariate and multivariate analyses results for T1G3 bladder cancer progression influencing factors. Cox univariate analysis displayed that tumor size (HR: 1.250; 95%CI: 1.093 - 3.185; p=0.018), multifocal (HR: 1.577; 95%CI: 1.196 - 2.604; p=0.026), past medical history (HR: 3.353; 95%CI: 2.074 - 6.082; p=0.031) and concomitant CIS (HR: 2.564; 95%CI: 1.202 - 4.954; p=0.010) were the influencing factors of T1G3 BCa progression. Furthermore, cox multivariate analysis also demonstrated that large size of tumor (HR: 1.546; 95%CI: 1.141 - 3.632; p=0.044), multifocal tumors (HR: 1.634; 95%CI: 1.068 - 3.732; p=0.038), recrudescent tumor (HR: 2.927; 95%CI: 1.269 - 4.973; p=0.042) and concomitant CIS (HR: 2.488; 95%CI: 1.104 - 5.464; p=0.012) were the independent adverse factors for T1G3 bladder cancer progression, which was in agreement with univariate analysis result.

### Kaplan-Meier survival analyses between clinicopathological factors and patient recurrence survival as well as progression survival

**Figure 1:**
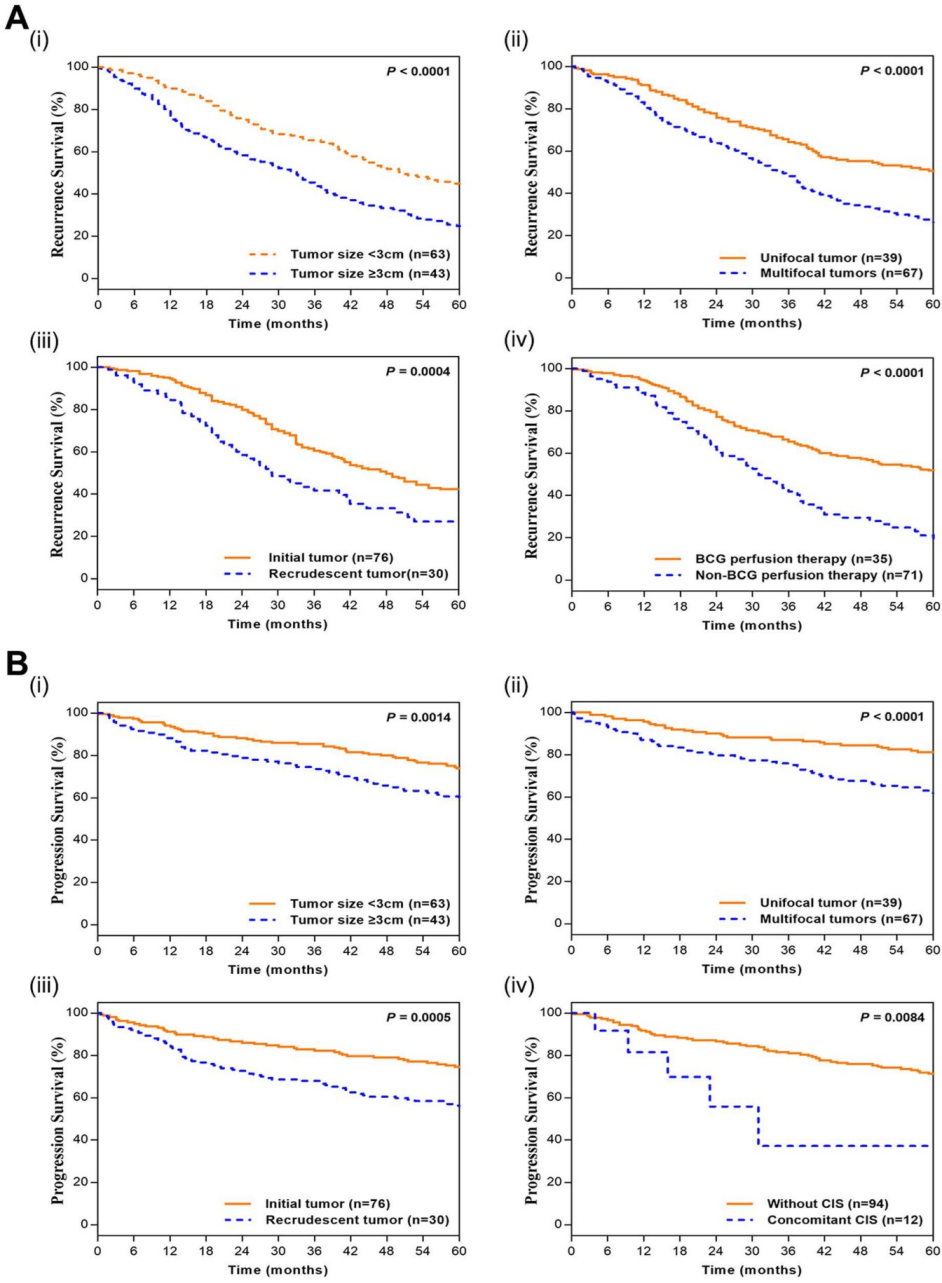
** Kaplan-Meier curves of T1G3 BCa patients' recurrence and progression survival. (A)** Kaplan-Meier curves of recurrence survival. i. tumor size; ii. Multifocal; iii. past medical history; iv. perfusion therapy. **(B)** Kaplan-Meier curves of progression survival. i. tumor size; ii. Multifocal; iii. past medical history; iv. concomitant CIS. P values were calculated with the log-rank test.

In the Kaplan-Meier survival analyses, compared to those T1G3 BCa patients with little size of tumor (<3 cm), unifocal tumor, initial tumor and BCG perfusion therapy, patients with large size of tumor (≥3 cm; HR=1.822; 95%CI: 1.413 - 2.350; p<0.0001), multifocal tumors (HR=2.038; 95%CI: 1.555 - 2.671; p<0.0001), recrudescent tumor (HR=1.801; 95%CI: 1.218 - 2.663; p=0.0004) and Non-BCG perfusion therapy (HR=2.423; 95%CI: 1.660 - 3.538; p<0.0001) had adverse recurrence survival. Moreover, patients with large size of tumor (≥3 cm; HR=1.712; 95%CI: 1.205 - 2.434; p=0.0014), multifocal tumors (HR=2.341; 95%CI: 1.639 - 3.346; p<0.0001), recrudescent tumor (HR=1.759; 95%CI: 1.273 - 2.432; p=0.0005) and concomitant CIS (HR=3.121; 95%CI: 0.674 - 14.440; p=0.0084) had adverse progression survival (Figure 1). It indicated that large size of tumor, multifocal tumors, recrudescent tumor, Non-BCG perfusion therapy and concomitant CIS led to adverse prognostic impact for T1G3 BCa patients, which was consistent with cox multivariate analyses.

### Construction of nomograms and calibration curves to predict recurrence and progression probability

**Figure 2:**
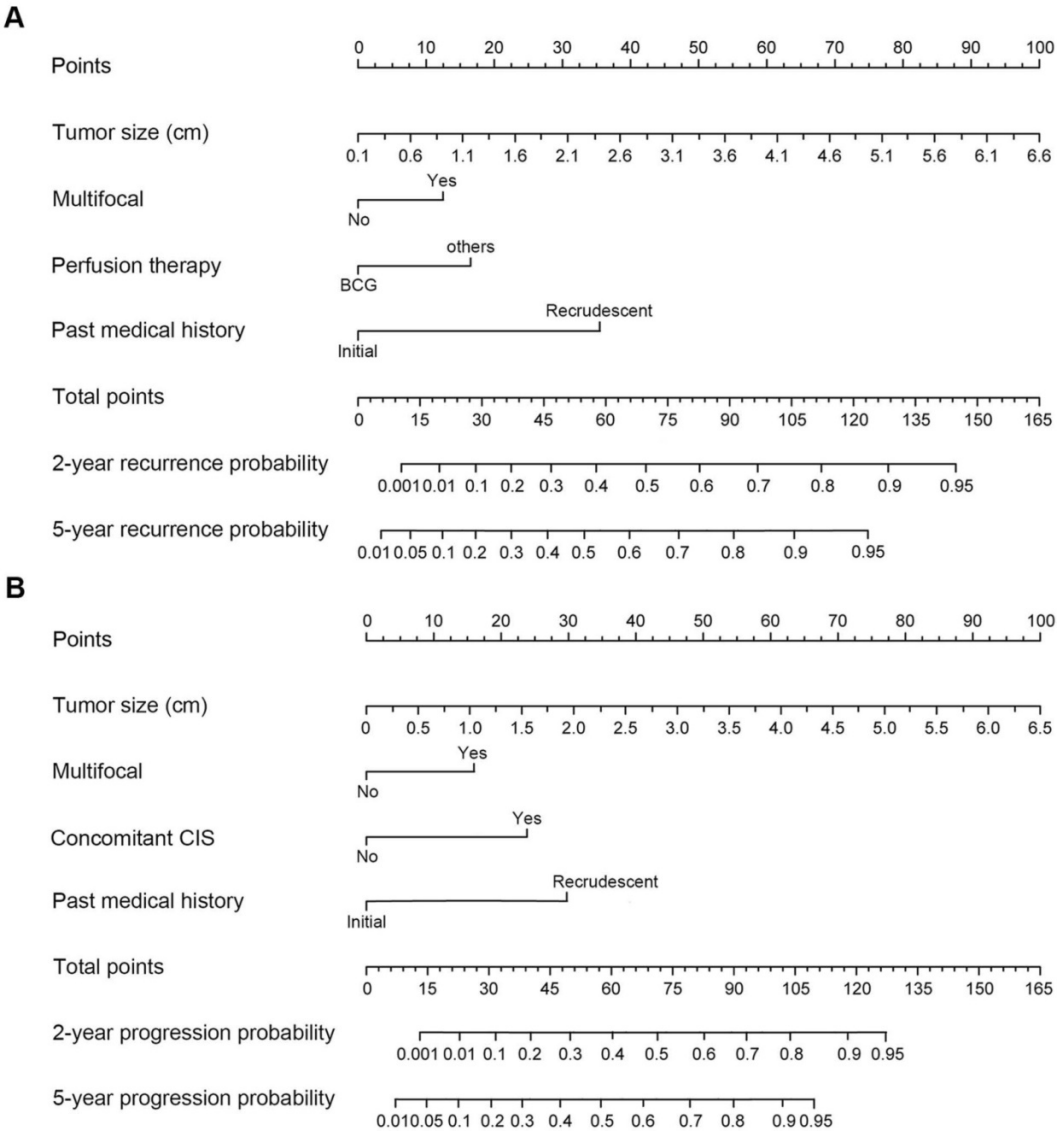
** The nomograms for recurrence and progression prediction of T1G3 BCa patients. (A)** Nomogram developed for recurrence probability. **(B)** the nomogram developed for progression probability. To estimate the risk of recurrence, the points for each variable were calculated by drawing a straight line from a patient's variable value to the axis labelled “Points”. The score sum is converted to a probability in the lowest axis.

**Figure 3:**
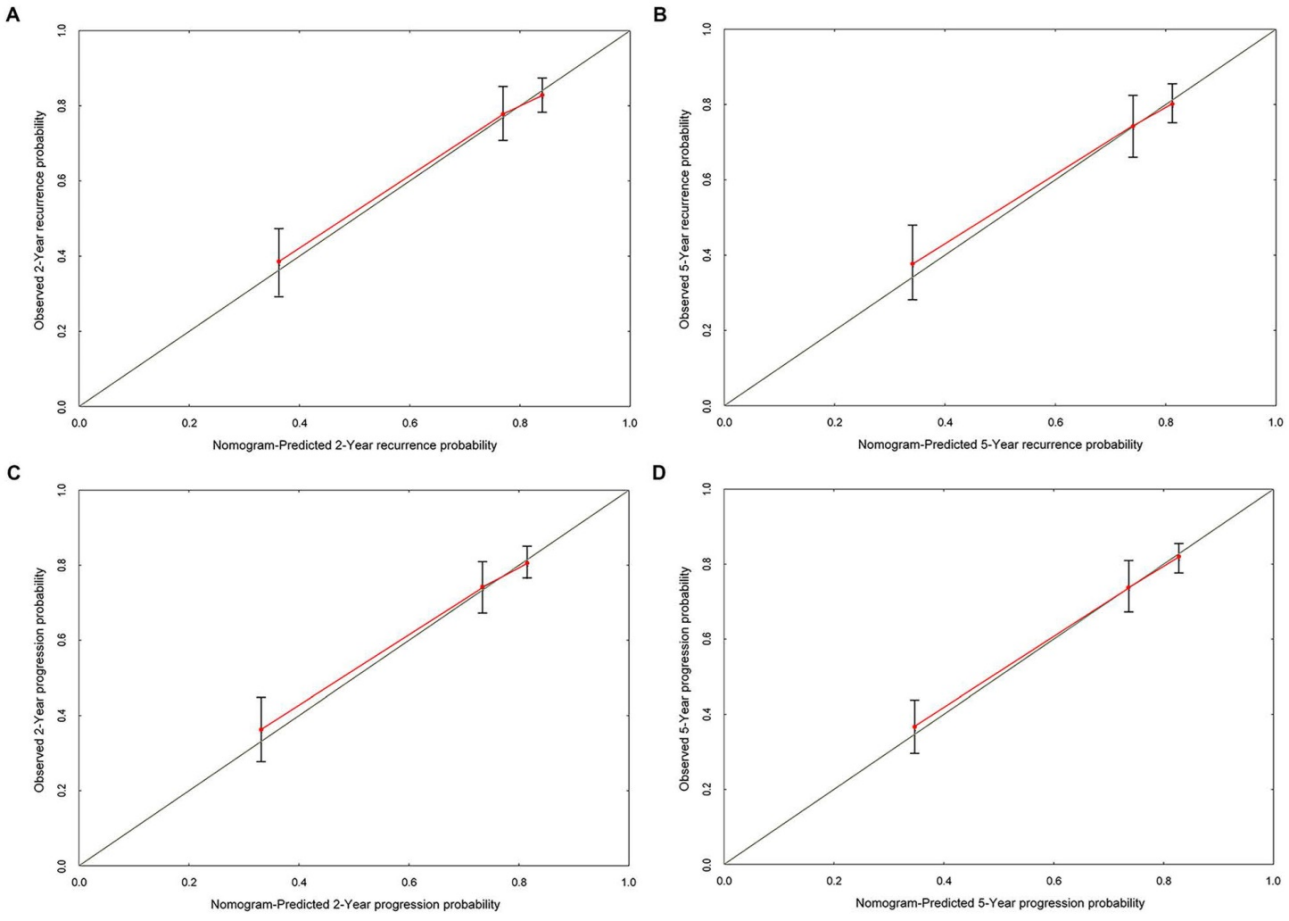
** The calibration curves developed for recurrence and progression prediction models. (A)** Calibration curve developed for 2-year recurrence prediction model. **(B)** Calibration curve developed for 5-year recurrence prediction model. **(C)** Calibration curve developed for 2-year progression prediction model. **(D)** Calibration curve developed for 5-year progression prediction model; the nomogram-predicted probability is plotted on the x-axis, and the actual probability is plotted on the y-axis.

Based on the cox multivariate analyses, the independent influencing factors such as tumor size, multifocal, past medical history, perfusion therapy and concomitant CIS could be included to generate the prediction nomograms of recurrence and progression. Nomograms were constructed to predict prognosis of each T1G3 BCa patient directly (Figure 2). The 2-year and 5-year recurrence probability as well as progression probability were able to be accurately calculated via the nomograms according to the information of each patient (tumor size, multifocal, past medical history, perfusion therapy and concomitant CIS). For example, a patient with a 3.3 cm size unifocal recrudescent tumor (without CIS) underwent BCG perfusion therapy, his total recurrence and progression points were 86 and 76, respectively, with an approximated 2-year recurrence probability of 58% and 5-year recurrence probability of 73%. Furthermore, his 2-year progression probability and 5-year progression probability were almost 55% and 68%, respectively. The calibration curves (Figure 3) displayed good agreement of the predicted probability with the actual observed probability for T1G3 BCa recurrence and progression, which indicated that these nomograms had great value of prediction.

### Evaluation of the prediction models for recurrence and progression

**Figure 4:**
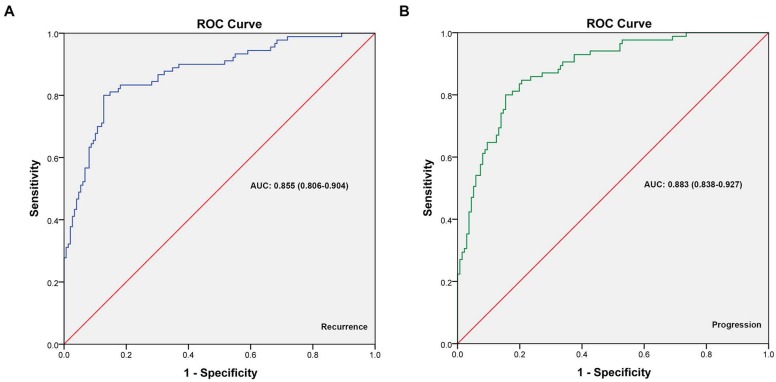
** The ROC curves developed for recurrence and progression prediction models. (A)** The ROC curve developed for 5-year recurrence prediction model. **(B)** The ROC curve developed for 5-year progression prediction model. The AUC of recurrence prediction model was 0.855 (95%CI: 0.806-0.904), and the progression prediction model was 0.883 (95%CI: 0.838-0.927).

ROC curves were generated for cox multivariate analyses to evaluate the value of the 5-year recurrence and progression prediction models (Figure 4). The AUC of recurrence prediction model was 0.855 (95%CI: 0.806-0.904), and the progression prediction model was 0.883 (95%CI: 0.838-0.927). It was been proved again that these prediction models had great value of prediction. To confirm the stability of the models, external data validations were performed, which was independently collected in another hospital. For 5-year recurrence prediction the sensitivity was 82.1% and the specificity was 77.3%; for 5-year progression prediction the sensitivity was 79.2% and the specificity was 81.1% (Supplementary table s1-s2).

Taken together, the results showed that recurrence and progression prediction models exhibit high accuracy and stability and is well generalized for other independent datasets.

## Discussion

According to statistics, there are 80,000 new cases of bladder cancer with 33,000 deaths in China every year 18. In the newly diagnosed cases, most of them are diagnosed with NMIBC, which is characterised by a high risk of recurrence and 17% progression to MIBC 5. Among these patients, T1G3 tumors have a higher propensity to recur and progress to MIBC, and its death rates as high as 34% 19. T1G3 tumor have been repeatedly reported as important risk factors for NMIBC recurrence and progression in a number of publications 4-7, on account of the poor prognosis and the high recurrence rate of T1G3 BCa patients, the optimal treatment choice for these patients remains controversial. It has been reported that the 5-year progression-free survival rate of T1G3 BCa patients treated with TURBT and intravesical BCG/chemotherapy was in the range of 60-80% 6, 8, while the T1G3 BCa patients with immediate radical cystectomy were reported with a 65-85% 5-year progression-free survival rate 5, 9, indicating that there was no much difference in prognosis between the two treatment approaches. In addition, Shahin, O. *et al.* reported that after treated with TURBT and intravesical BCG therapy, about 30% of T1G3 BCa patients never had recurred, and 30% of patients need defer radical cystectomy, while another third finally died of metastatic disease 10. A study based on 431 patients showed that the recurrence rate after a single epirubicin instillation was decreased by nearly half compared with intravesical instillation of water 20. Naya et al. reported that instillation of pirarubicin could reduce the risk of tumor recurrence in NMIBC patients with intermediate risk 21. In addition, intravesical administration of gemcitabine has an excellent toxicity profile and promising efficacy in NMIBC patients, which has been reported in several clinical trials 22-24. Because of the variable outcomes of T1G3 patients, the treatment options for T1G3 bladder cancer remains a challenge. And for this reason, recurrence and progression prediction is particularly important in T1G3 bladder cancer.

**Figure 5:**
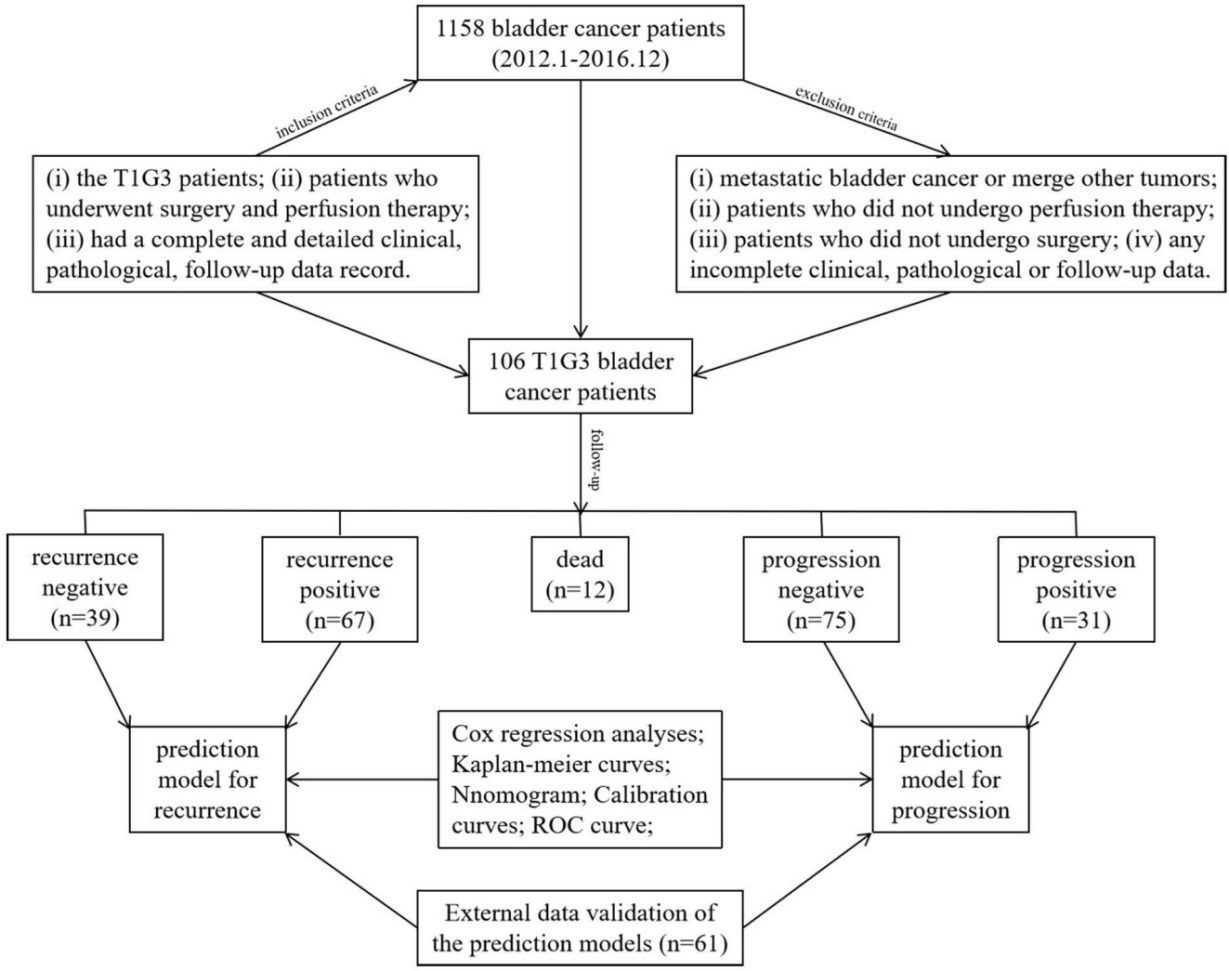
** The inclusion criteria and research process in this study.** A total of 1158 bladder cancer patients from January 2012 until December 2016 were involved in this study, and under the inclusion criteria 106 T1G3 bladder cancer patients were followed-up to establish the prediction models for recurrence and progression, the stability of prediction models were validated with an external cohort.

A total of 1158 BCa patients were recorded hospitalized in our center and 106 patients with T1G3 BCa of them (9.2%, 106/1158) were enrolled in this study according to the inclusion criteria and exclusion criteria. The patient follow-up protocol was performed and recurrence was identified in 67 cases (63.2%), progression was observed in 31 cases (29.2%), 17 cases (16.0%) were observed dead. In this study, we evaluated the recurrence and progression influencing factors in 106 T1G3 BCa patients, and established the prediction models for recurrence and progression, the stability of prediction models were validated with an external cohort (Figure 5).

Miller KD, *et al.* reported that the incidence of bladder cancer is about 4 times more frequent in men than in women 3, our date of 1158 patients with BCa showed the male-to-female ratio was 4.2:1, but the date of 106 T1G3 BCa was 2.7:1. Several studies pointed out that the reasons for this gender disparity in BCa maybe the lower prevalence of smoking among women and the higher exposure to carcinogens in men 25-26. Tobacco contains aromatic amines, which are known to cause bladder cancer, and these carcinogens usually are renally excreted to produce a carcinogenic effect on the whole urinary system. However, smoking history does not solely explain the difference risk of bladder cancer between sexes. Shiota M, *et al.* reported that androgen and androgen receptor signaling might play an important role in bladder cancer progression 27. Despite the higher incidence of male preponderance, it has been reported that female gender was a prognostic factor for worse cancer-specific survival following diagnosis with bladder cancer 25. Similarly, our results also showed that women with T1G3 BCa had higher recurrence rate (79.3% in female vs 57.1% in male) and higher progression rate (34.5% in female vs 27.3% in male). There was significant association between gender and recurrence, but no significant association in progression. Additionally, the cox analyses results showed gender was not an independent influencing factor for T1G3 BCa recurrence or progression (both p>0.05). More research is needed to explore the relationship between this gender disparity and T1G3 bladder cancer recurrence and progression.

Admittedly, smoking is recognized as the most important risk factor for bladder cancer, and estimated to account for 50% of tumors, the current smoking triples bladder cancer risk compared to never smoking 18-19. 59.4% (63/106) T1G3 BCa patients had the smoking history in our date, whereas no significant association was seen between smoking history and recurrence as well as progression (both p>0.05). Furthermore, smoking history was not identified as an independent risk factor for T1G3 BCa recurrence or progression in the cox analyses (both p>0.05).

Besides the gender and smoking history, the correlation analysis between recurrence and clinicopathological factors of T1G3 bladder urothelial carcinoma indicated T1G3 BCa recurrence was associated with old age (p=0.047), tumor size (p=0.031), multifocal (p=0.018), past medical history (p=0.024) and perfusion therapy (p=0.028). However, no significant association was observed between T1G3 bladder cancer recurrence and tumor location, concomitant CIS and operative method. In addition, the correlation analysis between progression and clinicopathological factors of T1G3 bladder urothelial carcinoma showed that T1G3 BCa progression was associated with tumor size (p=0.026), multifocal (p=0.017), past medical history (p=0.045) and concomitant CIS (p=0.044). In contrast, the other clinicopathological factors had no significant association with T1G3 BCa progression. In agreement with our conclusion, previous studies have identified some similar factors for BCa recurrence and progression, including concomitant CIS, multifocal tumors, lymphovascular invasion, female sex, older age and large size of tumor (>3 cm) 11-13. The EORTC risk tables and the CUETO scoring model were established to calculate the probability of recurrence and progression based on these factors 5, 14. However, most of these prediction models were based on different tumor stage and grade of NMIBC patients, and had a number of limitations for prediction of T1G3 patients with worse prognosis. D Andrea D, et al. 28 has developed a clinical decision-making tool (nomogram) to predict the progression to muscle-invasive disease in patients with pT1G3 bladder cancer. Nevertheless, the nomogram only applies to T1G3 patients who are treated with BCG, and the lymphovascular invasion (LVI) detection is not universal in the TURB period. In our study, cox analyses results displayed that large size of tumor (HR: 2.461; p=0.021), multifocal tumors (HR: 2.524; p<0.001), recrudescent tumor (HR: 3.069; p=0.009) were the independent risk factors for T1G3 BCa recurrence, meanwhile BCG perfusion therapy (HR: 0.642; p=0.012) was a protective factor for recurrence. Large size of tumor (HR: 1.546; p=0.044), multifocal tumors (HR: 1.634; p=0.038), recrudescent tumor (HR: 2.927; p=0.042) and concomitant CIS (HR: 2.488; p=0.012) were the independent risk factors for progression. As expected, our results demonstrated that BCG perfusion therapy could reduce recurrence rate of T1G3 BCa significantly, and there was no significant difference in prognosis between TURBT and partial cystectomy.

Then we constructed nomograms models to predict prognosis of each T1G3 BCa patient directly. The 2-year and 5-year recurrence probability as well as progression probability were able to be accurately calculated via the nomograms according to the information of each patient (tumor size, multifocal, past medical history, perfusion therapy and concomitant CIS). Calibration curves, ROC curves and external validation displayed that the nomograms had great value of prediction.

Of course, considering the effect of racial/ethnic differences, regional disparity in recurrence rate of BCa 29 and the limitation of small amount of data, multiple center data, more cases are needed for further study. We think that our prediction models of recurrence and progression risk could provide an evidence for clinical decision of many patients with T1G3 BCa, especially Chinese patients.

## Conclusion

Based on 106 patients with T1G3 BCa in our hospital, this study evaluated several recrudescent and progresses risk factors of T1G3 bladder urothelial carcinoma, indicating that large size of tumor, multifocal tumors, recrudescent tumor were the independent risk factors for T1G3 BCa recurrence, meanwhile BCG perfusion therapy was a protective factor for recurrence. Large size of tumor, multifocal tumors, recrudescent tumor and concomitant CIS were the independent risk factors for T1G3 BCa progression. Moreover, based on the cox regression analyses results, we established nomograms as prediction models to calculate the recurrence and progression probability of T1G3 BCa patients, calibration curves, ROC curves and external validation displayed that the nomograms had great value of prediction.

## Supplementary Material

Supplementary tables.

## Figures and Tables

**Table 1:** Follow-up results of 106 patients with T1G3 bladder cancer in development cohort.

Event	Case, n (%)
**Recurrence**	
No	39 (36.8)
Yes	67 (63.2)
**The first recurrence time (months)**	
≤12	29 (27.4)
>12	38 (35.8)
**Number of recurrence**	
Never	39 (36.8)
Once	38 (35.8)
Multiple times	29 (27.4)
**Progression**	
No	75 (70.8)
Yes	31 (29.2)
**The progression time (months)**	
≤12	11 (10.4)
>12	20 (18.9)
**Death**	
No	89 (84.0)
Yes	17 (16.0)
**The death time (months)**	
≤24	6 (5.7)
>24	11 (10.4)

**Table 2:** Clinical characteristics of enrolled patients in development cohort and validation cohort.

Variables	All patients (n=167)	Development cohort (n=106)	Validation cohort (n=61)	p value
**Gender, n (%)**				0.696
Male	123 (73.7)	77 (72.6)	46 (75.4)	
Female	44 (26.3)	29 (27.4)	15 (24.6)	
**Age/years, n (%)**				0.451
Average/Median (Range)	70.6±9.3/70	70.3±9.7/70	71.2±8.2/71	
	48-87	48-87	53-84	
<60	40 (24.0)	27 (25.5)	13 (21.3)	
60-69	50 (29.9)	33 (31.1)	17 (27.9)	
70-79	48 (28.7)	30 (28.3)	18 (29.5)	
≥80	29 (17.4)	16 (15.1)	13 (21.3)	
**Smoking history, n (%)**				0.716
No	66 (39.5)	43 (40.6)	23 (37.7)	
Yes	101 (60.5)	63 (59.4)	38 (62.3)	
**Tumor location, n (%)**				0.908
Vesical trigone	60 (35.9)	38 (35.8)	22 (36.1)	
Sidewall	69 (41.3)	45 (42.5)	24 (39.3)	
Anterior and posterior wall	22 (13.2)	13 (12.3)	9 (14.8)	
Others	16 (9.6)	10 (9.4)	6 (9.8)	
**Tumor size(cm), n (%)**				0.852
<1	58 (34.7)	36 (34.0)	22 (36.1)	
1-3	40 (24.0)	27 (25.5)	13 (21.3)	
≥3	69 (41.3)	43 (40.6)	26 (42.6)	
**Multifocal, n (%)**				0.743
No	63 (37.7)	39 (36.8)	24 (39.3)	
Yes	104 (62.3)	67 (63.2)	37 (60.7)	
**Past medical history, n (%)**				0.773
Initial	121 (72.5)	76 (77.7)	45 (73.8)	
Recrudescent	46 (27.5)	30 (28.3)	16 (26.2)	
**Concomitant CIS, n(%)**				0.520
No	150 (89.8)	94 (88.7)	56 (91.8)	
Yes	17 (10.2)	12 (11.3)	5 (8.2)	
**Perfusion therapy, n (%)**				0.639
BCG	53 (31.7)	35 (33.0)	18 (29.5)	
Others	114 (68.3)	71 (67.0)	43 (70.5)	
**Operative method, n (%)**				0.418
Partial cystectomy	24 (14.4)	17 (16.0)	7 (11.5)	
TURBT	143 (85.6)	89 (84.0)	54 (88.5)	

**Table 3:** Clinical characteristics of recurrence of T1G3 bladder urothelial carcinoma.

Variables	Recurrence negative (n=39)	Recurrence positive (n=67)	p value
Gender, n (%)			0.035
Male	33 (84.6)	44 (65.7)	
Female	6 (15.4)	23 (34.3)	
Age/years, n (%)			0.047
Average/Median (Range)	70.0±9.5/70	70.5±9.8/71	
	54-87	48-87	
<60	12 (30.8)	15 (22.4)	
60-69	13 (33.3)	20 (29.9)	
70-79	9 (23.1)	21 (31.3)	
≥80	5 (12.8)	11 (16.4)	
Smoking history, n (%)			0.192
No	19 (48.7)	24 (35.8)	
Yes	20 (51.3)	43 (64.2)	
Tumor location, n (%)			0.913
Vesical trigone	14 (35.9)	24 (35.8)	
Sidewall	16 (41.0)	29 (43.3)	
Anterior and posterior wall	5 (12.8)	8 (11.9)	
Others	4 (10.3)	6 (9.0)	
Tumor size(cm), n (%)			0.031
<1	21 (53.8)	15 (22.3)	
1-3	7 (17.9)	20 (29.9)	
≥3	11 (28.2)	32 (47.8)	
Multifocal, n (%)			0.018
No	20 (51.3)	19 (28.4)	
Yes	19 (48.7)	48 (71.6)	
Past medical history, n (%)			0.024
Initial	33 (84.6)	43 (64.2)	
Recrudescent	6 (15.4)	24 (35.8)	
Concomitant CIS, n(%)			0.561
No	36 (92.3)	58 (86.6)	
Yes	3 (7.7)	9 (13.4)	
Perfusion therapy, n (%)			0.028
BCG	18 (46.2)	17 (25.4)	
Others	21 (53.8)	50 (74.6)	
Operative method, n (%)			0.682
Partial cystectomy	7 (17.9)	10 (14.9)	
TURBT	32 (82.1)	57 (85.1)	

**Table 4:** Clinical characteristics of progression of T1G3 bladder urothelial carcinoma.

Variables	Progression negative (n=75)	Progression positive (n=31)	p value
**Gender, n (%)**			0.467
Male	56 (84.6)	21 (65.7)	
Female	19 (15.4)	10 (34.3)	
**Age/years, n (%)**			0.735
Average/Median (Range)	70.3±9.8/70	70.2±9.3/70	
	48-87	52-87	
<60	18 (24.0)	9 (29.0)	
60-69	25 (33.3)	8 (25.8)	
70-79	21 (28.0)	9 (29.0)	
≥80	11 (14.7)	5 (16.1)	
**Smoking history, n (%)**			0.802
No	31 (41.3)	12 (38.7)	
Yes	44 (58.7)	19 (61.3)	
**Tumor location, n (%)**			0.958
Vesical trigone	28 (37.3)	10 (32.3)	
Sidewall	31 (41.3)	14 (45.2)	
Anterior and posterior wall	9 (12.0)	4 (12.9)	
Others	7 (9.3)	3 (9.7)	
**Tumor size(cm), n (%)**			0.026
<1	30 (40.0)	6 (19.4)	
1-3	19 (25.3)	8 (25.8)	
≥3	26 (34.7)	17 (54.8)	
**Multifocal, n (%)**			0.017
No	33 (44.0)	6 (19.4)	
Yes	42 (56.0)	25 (80.6)	
**Past medical history, n (%)**			0.045
Initial	58 (77.3)	18 (58.1)	
Recrudescent	17 (22.7)	13 (41.9)	
**Concomitant CIS, n(%)**			0.044
No	70 (93.3)	24 (77.4)	
Yes	5 (6.7)	7 (22.6)	
**Perfusion therapy, n (%)**			0.054
BCG	29 (38.7)	6 (19.4)	
Others	46 (61.3)	25 (80.6)	
**Operative method, n (%)**			0.784
Partial cystectomy	13 (17.3)	4 (12.9)	
TURBT	62 (82.7)	27 (87.1)	

**Table 5:** Cox regression analyses for T1G3 bladder urothelial carcinoma recurrence.

Variables	Univariate analysis				Multivariate analysis		
	HR	95% CI	p value		HR	95% CI	p value
Gender (male/female)	0.940	0.822 - 1.016	0.163		-	-	-
Age (years)	1.135	0.861 - 1.443	0.415		-	-	-
Smoking history (yes/no)	1.047	0.974 - 1.201	0.256		-	-	-
Tumor location (vesical trigone/others)	1.011	0.868 - 1.136	0.817		-	-	-
Tumor size (cm)	2.173	1.412 - 3.368	0.014		2.461	1.358-3.975	0.021
Multifocal (yes/no)	1.627	1.128 - 3.095	0.035		2.524	1.510-4.139	<0.001
Past medical history (recrudescent/initial)	2.147	1.237 - 4.058	0.042		3.069	1.064-6.488	0.009
Concomitant CIS (yes/no)	1.146	0.895 - 1.714	0.119		-	-	-
Perfusion therapy (BCG/others)	0.768	0.341 - 0.922	0.048		0.642	0.289-0.864	0.012
Operative method (partial cystectomy/TURBT)	0.812	0.463 - 1.135	0.166		-	-	-

**Table 6:** Cox regression analyses for T1G3 bladder urothelial carcinoma progression.

Variables	Univariate analysis				Multivariate analysis		
	HR	95% CI	p value		HR	95% CI	p value
Gender (male/female)	0.945	0.675 - 1.458	0.383		-	-	-
Age (years)	1.122	0.749 - 1.689	0.414		-	-	-
Smoking history (yes/no)	1.263	0.808 - 1.751	0.328		-	-	-
Tumor location (vesical trigone/others)	1.106	0.991 - 1.257	0.915		-	-	-
Tumor size (cm)	1.250	1.093 - 3.185	0.018		1.546	1.141 - 3.632	0.044
Multifocal (yes/no)	1.577	1.196 - 2.604	0.026		1.634	1.068 - 3.732	0.038
Past medical history (recrudescent/initial)	3.353	2.074 - 6.082	0.031		2.927	1.269 - 4.973	0.042
Concomitant CIS (yes/no)	2.564	1.202 - 4.954	0.010		2.488	1.104 - 5.464	0.012
Perfusion therapy (BCG/others)	0.818	0.405 - 1.233	0.214		-	-	-
Operative method (partial cystectomy/TURBT)	0.769	0.467 - 1.162	0.193		-	-	-

## Abbreviations

AUC: Areas Under the Curves
BCa: Bladder Cancer
BCG: Bacillus Calmette-Guérin
CIS: Carcinoma in Situ
LVI: Lymphovascular Invasion
MIBC: Muscle-Invasive Bladder Cancer
NMIBC: Non-Muscle-Invasive Bladder Cancer
ROC: Receiver Operating Characteristic
TURBT: Transurethral Resection of Bladder Tumor
