# Supplementary material for: Establishing the prediction models for recurrence and progression of T1G3 bladder urothelial carcinoma
Source: J Cancer. 2019 Oct 11;10(24):5891–902. doi: 10.7150/jca.35866 (PMC6856570; doi:10.7150/jca.35866)
Supplement: Supplementary file 1 — Supplementary tables. [file jcav10p5891s1.pdf]

Establishing the prediction models for recurrence and progression of T1G3 bladder urothelial carcinoma

Supplementary Tables

Supplementary Table S1. External data validation of the prediction model for recurrence.

		2-year recurrence (Validation cohort)		Total			5-year recurrence (Validation cohort)		Total
		No	Yes				No	Yes	
2-year recurrence (Prediction model)	No	28	5	33	5-year recurrence (Prediction model)	No	17	5	22
	Yes	6	22	28		Yes	7	32	39
Total		34	27	61	Total		24	37	61

2-year recurrence

Sensitivity =22/(22+6)=78.6%

Specificity =28/(28+5)=84.8%

5-year recurrence

Sensitivity =32/(32+7)=82.1%

Specificity =17/(17+5)=77.3%

Supplementary Table S2. External data validation of the prediction model for progression.

		2-year progression (Validation cohort)		Total			5-year progression (Validation cohort)		Total
		No	Yes				No	Yes	
2-year progression (Prediction model)	No	41	7	48	5-year progression (Prediction model)	No	30	7	37
	Yes	3	10	13		Yes	5	19	24
Total		44	17	61	Total		35	26	61
